# Graded RhoA GTPase Expression in Treg Cells Distinguishes Tumor Immunity From Autoimmunity

**DOI:** 10.3389/fimmu.2021.726393

**Published:** 2021-10-15

**Authors:** Khalid W. Kalim, Jun-Qi Yang, Vishnu Modur, Phuong Nguyen, Yuan Li, Yi Zheng, Fukun Guo

**Affiliations:** Division of Experimental Hematology and Cancer Biology, Children’s Hospital Medical Center, and Department of Pediatrics, University of Cincinnati College of Medicine, Cincinnati, OH, United States

**Keywords:** RhoA, Treg cells, effector T cells, autoimmunity, tumor immunity

## Abstract

RhoA of the Rho GTPase family is prenylated at its C-terminus. Prenylation of RhoA has been shown to control T helper 17 (Th17) cell-mediated colitis. By characterizing T cell-specific RhoA conditional knockout mice, we have recently shown that RhoA is required for Th2 and Th17 cell differentiation and Th2/Th17 cell-mediated allergic airway inflammation. It remains unclear whether RhoA plays a cell-intrinsic role in regulatory T (Treg) cells that suppress effector T cells such as Th2/Th17 cells to maintain immune tolerance and to promote tumor immune evasion. Here we have generated Treg cell-specific RhoA-deficient mice. We found that homozygous RhoA deletion in Treg cells led to early, fatal systemic inflammatory disorders. The autoimmune responses came from an increase in activated CD4^+^ and CD8^+^ T cells and in effector T cells including Th17, Th1 and Th2 cells. The immune activation was due to impaired Treg cell homeostasis and increased Treg cell plasticity. Interestingly, heterozygous RhoA deletion in Treg cells did not affect Treg cell homeostasis nor cause systemic autoimmunity but induced Treg cell plasticity and an increase in effector T cells. Importantly, heterozygous RhoA deletion significantly inhibited tumor growth, which was associated with tumor-infiltrating Treg cell plasticity and increased tumor-infiltrating effector T cells. Collectively, our findings suggest that graded RhoA expression in Treg cells distinguishes tumor immunity from autoimmunity and that rational targeting of RhoA in Treg cells may trigger anti-tumor T cell immunity without causing autoimmune responses.

## Introduction

After maturation in the thymus, T lymphocytes migrate to the peripheral lymphoid organs where they are maintained as naïve T cells. Upon antigen recognition, naïve T cells differentiate to effector T cells such as CD4^+^IFN-γ^+^ T helper 1 (Th1) cells, CD4^+^ IL-17^+^ Th17 cells, CD4^+^IL-4^+^ Th2 cells, and CD8^+^IFN-γ^+^ cytotoxic T cells (CTL) (1). Effector T cells play an important role in preventing pathogen infection as well as tumor development (2). However, abnormal effector T cell responses may cause autoimmune diseases (3).

Regulatory T (Treg) cells that are also developed in the thymus function to maintain immune tolerance, primarily by inhibiting effector T cells (4). Defects in Treg cell development, homeostasis and/or function can lead to autoimmunity (5, 6). In the tumor microenvironment (TME), Treg cells provide an important immune suppressive mechanism to promote tumor growth (7). Depletion of Treg cells may thus benefit cancer patients (8). However, systemic removal of Treg cells likely causes autoimmune responses (8). It has recently been suggested that cancer may be treated by induction of Treg cell plasticity that in general is reflected by loss of stable expression of Foxp3, the signature transcription factor of Treg cells, effector T cell reprogramming, and/or impairment of Treg cell function (7, 9–11).

RhoA belongs to the Rho family small GTPases of Ras superfamily. Like other GTPases, RhoA is activated upon binding to GTP and inactivated upon binding to GDP. RhoA activity is also regulated by prenylation at its C-terminus (12). RhoA is well-known to play an important role in actin cytoskeleton organization, cell adhesion, migration, proliferation, and survival (13–17). In T cells, RhoA modulates T cell polarization, thymocyte development and adhesion, and thymic egress (18–25). Prenylation of RhoA by PGGT1B has been shown to control Th17 cell-mediated colitis (26). By conditional knockout of *RhoA* in pan T cells, we have recently shown that RhoA is required for Th2 and Th17 cell differentiation (27, 28). Interestingly, while *RhoA* deletion dampens Th2 and Th17 cell differentiation, it does not affect Th1 cell differentiation (27). In addition, it is seemingly that *RhoA* deletion in pan T cells does not impair Treg cell development and homeostasis (28). Nonetheless, it remains unclear whether RhoA plays a cell-intrinsic role in Treg cells. In this study, we generated Treg cell-specific *RhoA* knockout mice. We found that homozygous *RhoA* deletion in Treg cells reduced peripheral Treg cell frequencies, induced Treg cell plasticity, and caused early, fatal inflammatory diseases, suggesting that RhoA is important for maintenance of Treg cell homeostasis and fitness and for controlling autoimmunity. In contrast, heterozygous *RhoA* deletion in Treg cells did not affect Treg cell frequencies nor cause inflammatory diseases. However, *RhoA* heterozygosity also induced Treg cell plasticity. Importantly, heterozygous deletion of *RhoA* activated anti-tumor T cell immunity. These findings suggest that graded RhoA expression in Treg cells distinguishes tumor immunity from autoimmunity.

## Materials and Methods

### Mice

*Foxp3^YFP-Cre^* mice were purchased from Jackson laboratories (Cat # 016959, RRID : IMSR_ JAX: 016959). *RhoA^Flox/Flox^* mice were generated as described previously (29). *RhoA^Flox/Flox^* mice were bred with *Foxp3^YFP-Cre^* mice in our animal facility to generate *RhoA^Flox/+^Foxp3^YFP-Cre^*, *RhoA^Flox/Flox^Foxp3^YFP-Cre^* and *RhoA^Flox/Flox^Foxp3^YFP-Cre/+^* mice. All mice were housed under specific pathogen-free conditions in the animal facility at the Cincinnati Children’s Hospital Research Foundation in compliance with the Cincinnati Children’s Hospital Medical Center Animal Care and Use Committee protocols.

### Tumor Cell Lines

Mouse colon adenocarcinoma GFP-tagged tumor cell line MC38 was generously provided by Dr. Joseph Palumbo at the Cincinnati Children’s Hospital Medical Center. The tumor cell line was grown in DMEM with 10% fetal calf serum (FCS). Regular mycoplasma testing was carried out on all cells maintained in culture.

### Tumor Growth Studies

Eight hundred thousand MC38 tumor cells were injected subcutaneously in 100 µl PBS into one of the flanks of the indicated mice. Tumor volumes were measured every two to three days after tumor became visible and calculated as V = (length × width^2^) x 0.50.

### Preparation of Single-Cell Suspensions, Antibody Staining, and Flow Cytometry

Spleens were mashed with a syringe plunger and passed through 40 µm cell strainer, followed by treatment with red blood cell lysis buffer (Cat # 555899, BD Biosciences). Tumors were minced into small fragments and treated with 1.5 mg/ml collagenase IV (Cat # C5138, Sigma) for 30 min at 37°C under agitation. The digested tumor tissue was then filtered through a 70 μm cell strainer and centrifuged at 1500 rpm at 4°C for 5 min. The pellets were dissolved in 8 ml of 40% Percoll and slowly layered over 5 ml of 80% Percoll in a 15 ml falcon tube. The falcon tube was then centrifuged at 2000 rpm at 4°C for 20 min, stopping without brakes. Cells at the interface between 40% Percoll and 80% Percoll were carefully removed and washed twice with complete RPMI T-cell medium. The cells from spleens and tumors were re-stimulated with PMA and Ionomycin for 5 hrs in the presence of Golgi plug for the last 4 hrs, and then subjected to antibody staining. Cell surface proteins were stained for 20 min at 4°C with the following antibodies: PD-1 (Cat # 12-9985-81, Clone J43, RRID : AB_466294, eBioscience), GITR (Cat # 25-5874-80, Clone DTA-1, RRID : AB_10544396, eBioscience), CTLA-4 (Cat # 12-1522-82, Clone UC10-4B9, RRID : AB_465879, eBioscience), CD4 (Cat # 48-0042-82, Clone RM4-5, RRID : AB_1272194, eBioscience), CD8α (Cat # 25-0081-82, Clone 53-6.7, RRID : AB_469584, eBioscience), CD62L (Cat # 12-0621-82, clone MEL-14, RRID: AB_465721, eBioscience), and CD44 (Cat # 17-0441-82, clone IM7, RRID: AB_469390, eBioscience). Intracellular proteins were stained for 60 min at room temperature after permeabilization and fixation with BD Cytofix/Cytoperm Plus (Cat # 555028, BD Biosciences) using the following antibodies: IL-4 (Cat # 12-7041-82, Clone 11B11, RRID : AB_466156, eBioscience), IL-17A (Cat # 559502, Clone TC11-18H10, RRID : AB_397256, BD Pharmingen), IFN-γ (Cat # 505810, 505826, Clone XMG1.2, RRID : AB_315404, RRID : AB_2295770, BioLegend), Foxp3 (Cat # 17-5773-82, Clone FJK-16s, vRRID : AB_469457, eBioscience), RORγT (Cat # 12-6988-82, Clone AFKJS-9, RRID : AB_1834470, eBioscience), T-bet (Cat # 45-5825-82, clone 4B10, RRID : AB_953657, eBioscience), and GATA-3 (Cat # 25-9966-42, Clone TWAJ, RRID : AB_2573568, eBioscience). The stained cells were analyzed by BD LSRII, FACSCanto, or LSRFortessa flow cytometers. Data were analyzed with BD FACSDiva.

For cell apoptosis assay, freshly isolated splenocytes were immunolabeled with anti-CD4, anti-Foxp3, and anti-active Caspase 3 (Cat # 559341, Clone C92-605, RRID : AB_397234, BD Pharmingen) followed by flow cytometry.

For cell proliferation assay, mice were injected intraperitoneally with 500 mg 5-bromo-29-deoxyuridine (BrdU). Two hours after injection, splenocytes were isolated and immunolabeled with anti-CD4 and anti-Foxp3 antibodies and BrdU incorporation was analyzed by a BrdU Flow kit per the manufacturer’s protocol (Cat # 552598, BD Pharmingen).

For *in vitro* Treg cell suppressive activity assay, splenic Treg cells were obtained by magnetic-activated cell sorting of CD4^+^ T cells (Cat # 130-117-043, Miltenyi) followed by flow cytometry sorting of CD4^+^YFP^+^ Treg cells. The purified Treg cells were activated by anti-CD3/CD28 Dynabeads Cat # 11456D, ThermoFisher) and IL-2 for 24 hrs. CD4^+^ naïve T cells were obtained by using a naïve T cell isolation kit (Cat # 130-104-453, Miltenyi) and labelled with CFSE (5 µM). The pre-activated Treg cells were mixed with the CFSE-labelled T cells at 1:1 ratio and cultured for 96 hrs in the presence of anti-CD3/CD28 Dynabeads. CFSE dilution was examined by flow cytometry.

### Histopathological Analysis

Tissues were sectioned, fixed in 4% formaldehyde solution, embedded in paraffin, and stained with H&E. The sections were analyzed by light microscopy from Fisher Scientific Moticam at 20X magnification at room temperature. The images were acquired by using the software Motic Images Plus 2.0 (30).

### Statistical Analysis

Mouse survival rate was analyzed by log-rank (Mantel–Cox) test. Tumor growth was analyzed by two-way ANOVA. All of the other statistics were performed with the two-tailed Student t-test. Data were expressed as mean ± SD. p < 0.05 was considered significant.

## Results

### Homozygous *RhoA* Deletion in Treg Cells Causes Systemic Inflammatory Disorders

**Figure 1:**
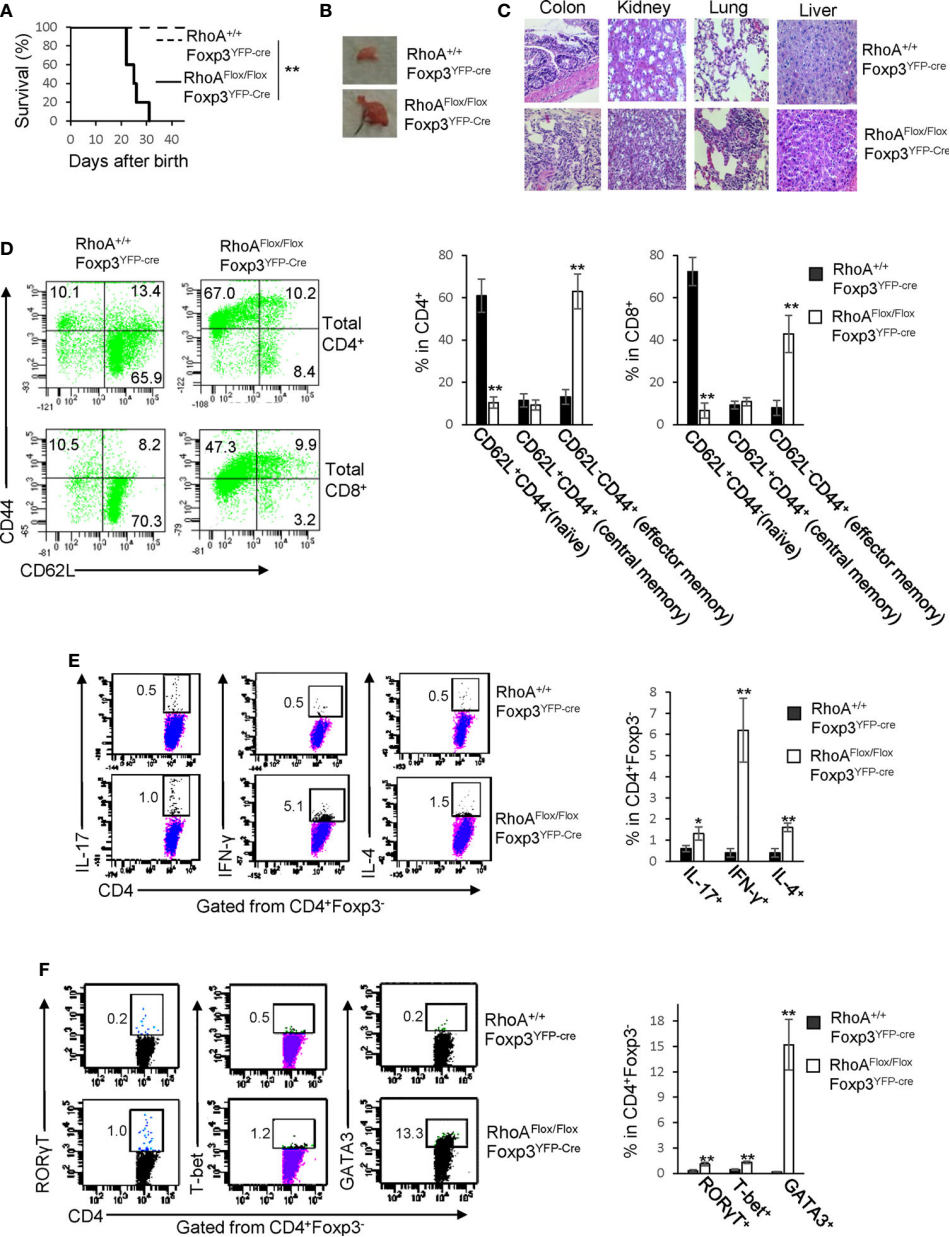
Homozygous *RhoA* deletion in Treg cells leads to early, fatal spontaneous inflammatory disorders. **(A)** Survival outcome of RhoA^+/+^Foxp3^YFP-Cre^ and RhoA^Flox/Flox^Foxp3^YFP-Cre^ mice. Results were analyzed with a log-rank (Mantel–Cox) test and expressed as Kaplan–Meier survival curves. **(B)** Image of lymphadenopathy in RhoA^Flox/Flox^Foxp3^YFP-Cre^ mice. Inguinal lymph nodes are shown. **(C)** Images of H&E staining of the indicated organs from RhoA^+/+^Foxp3^YFP-Cre^ and RhoA^Flox/Flox^Foxp3^YFP-Cre^ mice (original magnification X 400). **(D)** Left, representative flow cytogram of CD44 and CD62L staining in CD4^+^ and CD8^+^ cells from the spleen of RhoA^+/+^Foxp3^YFP-Cre^ and RhoA^Flox/Flox^Foxp3^YFP-Cre^ mice. The numbers indicate percentages of CD44^+^, CD44^+^CD62L^+^, and CD62L^+^ cells. Right, average percentages of CD44^+^, CD44^+^CD62L^+^, and CD62L^+^ cells. **(E)** Left, representative flow cytogram of IL-17, IFN-γ, and IL-4 staining in CD4^+^Foxp3^-^ cells from the spleen of RhoA^+/+^Foxp3^YFP-Cre^ and RhoA^Flox/Flox^Foxp3^YFP-Cre^ mice. The numbers indicate percentages of IL-17^+^, IFN-γ^+^, and IL-4^+^ cells. Right, average percentages of IL-17^+^, IFN-γ^+^, and IL-4^+^ cells. **(F)** Left, representative flow cytogram of RORγT, T-bet and GATA3 staining in CD4^+^Foxp3^-^ cells from the spleen of RhoA^+/+^Foxp3^YFP-Cre^ and RhoA^Flox/Flox^Foxp3^YFP-Cre^ mice. The numbers indicate percentages of RORγT ^+^, T-bet^+^, and GATA3^+^ cells. Right, average percentages of RORγT ^+^, T-bet^+^, and GATA3^+^ cells. **(A, D–F)** n = 3-5 mice. Data are representative of two independent experiments. Error bars indicate SD. *p < 0.05, **p < 0.01. **(B, C)** Data are representative of 3 mice.

*RhoA^Flox/Flox^Foxp3^YFP-Cre^* mice harboring Treg cell-specific homozygous *RhoA* deletion were small in size, lacked mobility, and developed a hunched posture and skin ulceration (Data not shown). The mice died within ∼3-5 weeks after birth (**Figure 1A**). Moreover, the mice displayed lymphadenopathy (**Figure 1B**) and massive leukocyte infiltration and/or distorted architecture in a variety of organs, particularly in the colon, kidney, and lung (**Figure 1C**). In line with these phenotypes, *RhoA^Flox/Flox^Foxp3^YFP-Cre^* mice contained more effector memory T cells (CD62L^-^CD44^+^) in their CD4^+^ and CD8^+^ compartments (**Figure 1D**). Furthermore, the mice showed an increase in effector T cells including IL-17-producing Th17, IFN-γ-producing Th1, and IL-4-producing Th2 cells (**Figure 1E**). Consistently, RORγT, T-bet and GATA3, signature transcriptional factors for Th17, Th1 and Th2 cells, respectively, were increased in CD4^+^ cells from *RhoA^Flox/Flox^Foxp3^YFP-Cre^* mice (**Figure 1F**). These findings suggest that homozygous *RhoA* depletion in Treg cells leads to systemic autoimmune responses.

### Homozygous *RhoA* Deletion in Treg Cells Impairs Treg Cell Homeostasis and Induces Treg Cell Plasticity

**Figure 2:**
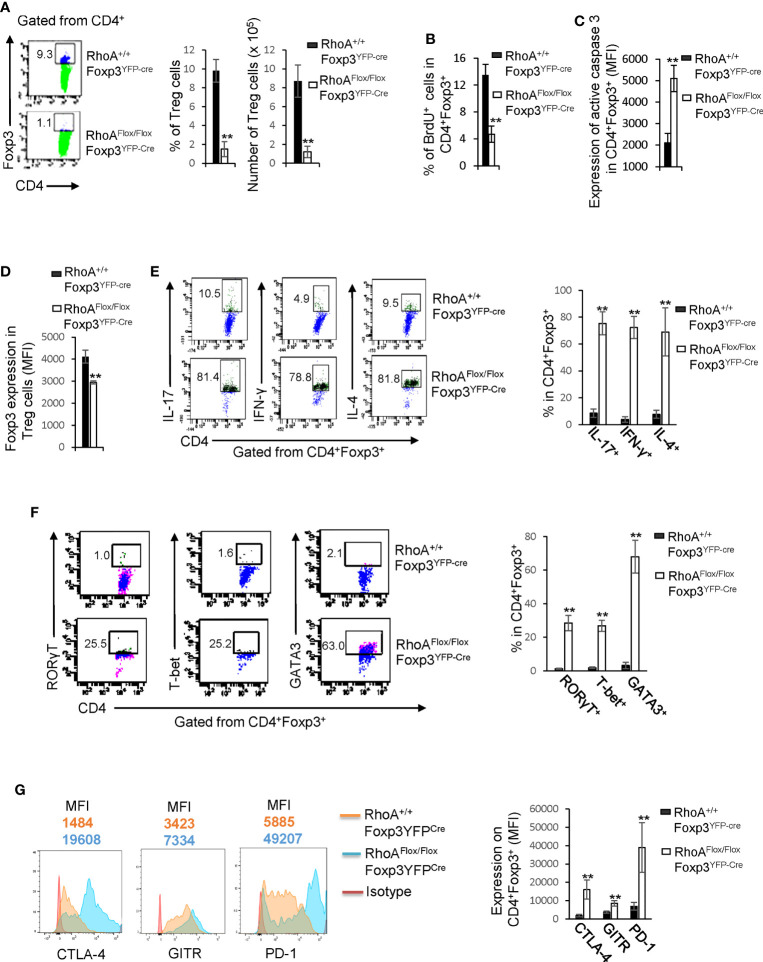
Homozygous *RhoA* deletion in Treg cells dampens Treg cell homeostasis and induces Treg cell plasticity. **(A)** Left, representative flow cytogram of Foxp3 staining in CD4^+^ cells from the spleen of RhoA^+/+^Foxp3^YFP-Cre^ and RhoA^Flox/Flox^Foxp3^YFP-Cre^ mice. The numbers indicate percentages of CD4^+^Foxp3^+^ Treg cells. Middle, average percentages of CD4^+^Foxp3^+^ Treg cells. Right, average numbers of CD4^+^Foxp3^+^ Treg cells. **(B)** Treg cell proliferation. Percentages of CD4^+^Foxp3^+^ Treg cells incorporated with BrdU are shown. **(C)** Treg cell apoptosis. The expression levels (MFI: Mean fluorescence intensity) of active caspase 3 in CD4^+^Foxp3^+^ Treg cells are shown. **(D)** The expression levels of Foxp3 in Treg cells. **(E)** Left, representative flow cytogram of IL-17, IFN-γ, and IL-4 staining in CD4^+^Foxp3^+^ Treg cells. The numbers indicate percentages of IL-17^+^, IFN-γ^+^, and IL-4^+^ Treg cells. Right, average percentages of IL-17^+^, IFN-γ^+^, and IL-4^+^ Treg cells. **(F)** Left, representative flow cytogram of RORγT, T-bet and GATA3 staining in CD4^+^Foxp3^+^ Treg cells. The numbers indicate percentages of RORγT ^+^, T-bet^+^, and GATA3^+^ Treg cells. Right, average percentages of RORγT ^+^, T-bet^+^, and GATA3^+^ Treg cells. **(G)** Left, representative histogram of the expression levels of CTLA-4, GITR and PD-1 in CD4^+^Foxp3^+^ Treg cells. The numbers above the graphs indicate MFI. Right, average MFI of CTLA-4, GITR and PD-1 in CD4^+^Foxp3^+^ Treg cells. n = 3 mice. Data are representative of two independent experiments. Error bars indicate SD. **p < 0.01.

We postulated that the systemic inflammatory disorders in *RhoA^Flox/Flox^Foxp3^YFP-Cre^* mice resulted from defects in Treg cells. Indeed, Treg cells were significantly decreased in *RhoA^Flox/Flox^Foxp3^YFP-Cre^* mice (**Figure 2A**). The loss of Treg cells was associated with decreased proliferation and increased apoptosis, as revealed by less BrdU^+^ and more active Caspase-3-expressing Treg cells in *RhoA^Flox/Flox^Foxp3^YFP-Cre^* mice than that in *RhoA^+/+^Foxp3^YFP-Cre^* mice (**Figures 2B, C**). Treg cells harboring homozygous *RhoA* depletion became plastic, as they showed reduced Foxp3 expression (**Figure 2D**) and increased effector T cell reprogramming, that is, more Treg cells producing effector T cell cytokines IL-17, IFN-γ, and IL-4 (**Figure 2E**) and transcription factors RORγT, T-bet, and GATA3 (**Figure 2F**). Moreover, *RhoA^Flox/Flox^Foxp3^YFP-Cre^* Treg cells upregulated their functional markers CTLA-4, GITR and PD-1 (**Figure 2G**).

**Figure 3:**
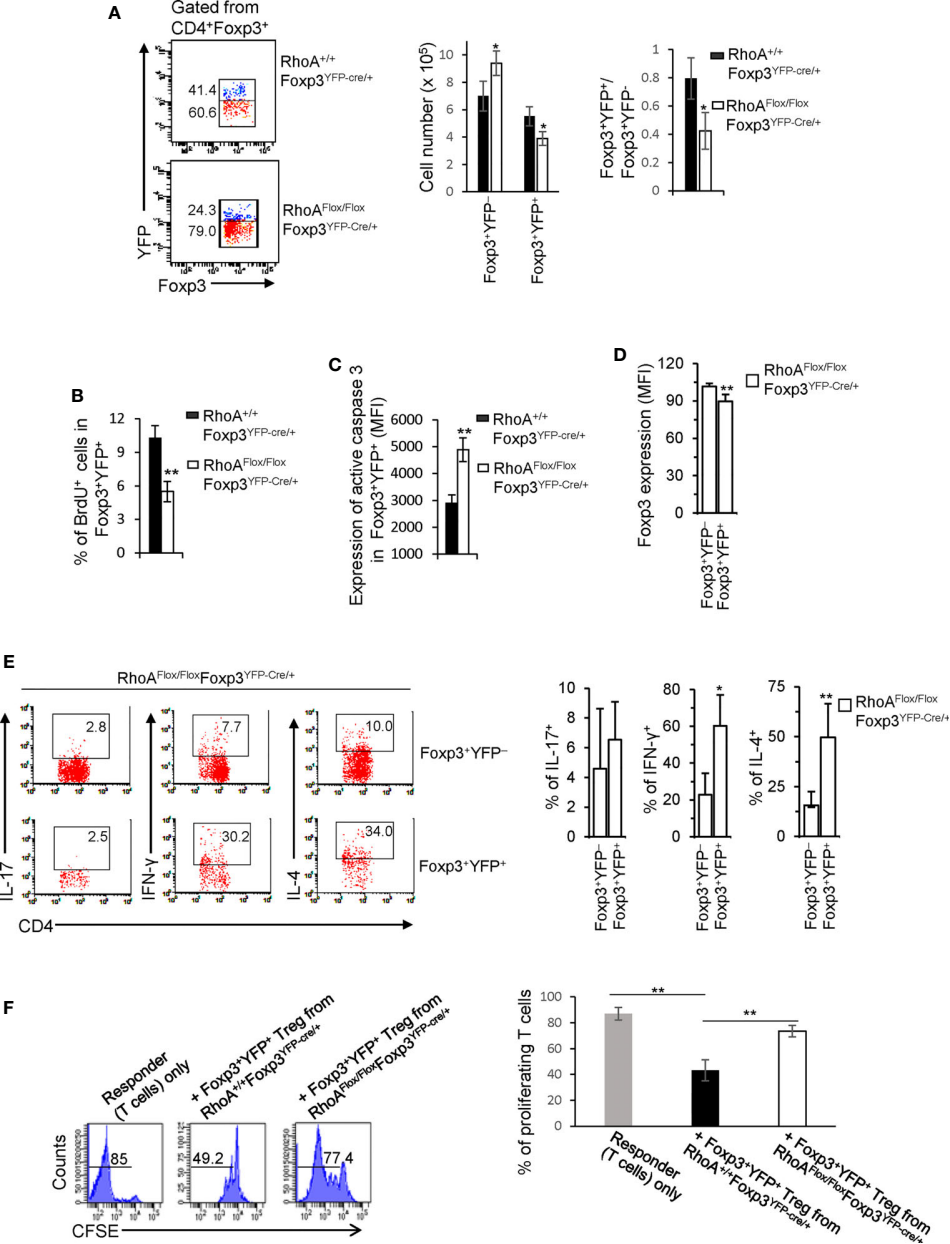
Treg cells bearing homozygous *RhoA* deletion are plastic and outcompeted by Treg cells bearing intact RhoA in RhoA^Flox/Flox^Foxp3^YFP-Cre/+^ female mice. **(A)** Left, representative flow cytogram of Foxp3^+^YFP^+^ and Foxp3^+^YFP^-^ cells in CD4^+^Foxp3^+^ Treg cells from the spleen of RhoA^+/+^Foxp3^YFP-Cre/+^ and RhoA^Flox/Flox^Foxp3^YFP-Cre/+^ female mice. The numbers indicate percentages of Foxp3^+^YFP^+^ and Foxp3^+^YFP^-^ cells. Middle, cell numbers of Foxp3^+^YFP^+^ and Foxp3^+^YFP^-^ cells. Right, the ratio of percentages of Foxp3^+^YFP^+^ versus percentages of Foxp3^+^YFP^-^ cells. **(B)** Foxp3^+^YFP^+^ Treg cell proliferation. **(C)** Foxp3^+^YFP^+^ Treg cell apoptosis. **(D)** The expression levels (MFI: Mean fluorescence intensity) of Foxp3 in Foxp3^+^YFP^+^ and Foxp3^+^YFP^-^ cells from the spleen of RhoA^Flox/Flox^Foxp3^YFP-Cre/+^ female mice. **(E)** Left, representative flow cytogram of IL-17, IFN-γ, and IL-4 staining in CD4^+^Foxp3^+^YFP^+^ and CD4^+^Foxp3^+^YFP^-^ cells from the spleen of RhoA^Flox/Flox^Foxp3^YFP-Cre/+^ female mice. The numbers indicate percentages of IL-17^+^, IFN-γ^+^, and IL-4^+^ cells. Right, average percentages of IL-17^+^, IFN-γ^+^, and IL-4^+^ cells. **(F)** *In vitro* Treg suppressive activity. Left, representative flow cytogram of CFSE dilution in T cells (responder). The numbers above the gating are percentages of cells low for CFSE, representing proliferating T cells. Right, average percentages of cells low for CFSE. Foxp3^+^YFP^+^ Treg cells were obtained by flow cytometry sorting of CD4^+^YFP^+^ cells from the spleen of RhoA^+/+^Foxp3^YFP-Cre/+^ and RhoA^Flox/Flox^Foxp3^YFP-Cre/+^ female mice. Error bars indicate SD of 3 mice **(A–E)** or triplicates **(F)**. Data are representative of two independent experiments. *p < 0.05, **p < 0.01.

It is known that inflammation may induce Treg cell plasticity (31). In this context, the plasticity of *RhoA^Flox/Flox^Foxp3^YFP-Cre^* Treg cells could be attributed to the inflammatory disorders in *RhoA^Flox/Flox^Foxp3^YFP-Cre^* mice. To test this, we analyzed *RhoA^Flox/Flox^Foxp3^YFP-Cre^*^/+^ female mice that are heterozygous for Foxp3^YFP-Cre^ and did not show overt inflammation (data not shown). Because of the X chromosome-linked nature of and random X chromosome inactivation by Foxp3^YFP-Cre^ knock-in transgene, Foxp3^YFP-Cre/+^ female mice are expected to maintain ~50% of Foxp3^+^YFP^+^ Treg cells and ~50% of Foxp3^+^YFP^-^ Treg cells (30). In support, *RhoA^+/+^Foxp3^YFP-Cre^*^/+^ female mice exhibited ~ 0.80:1 ratio of Foxp3^+^YFP^+^: Foxp3^+^YFP^-^ cells (**Figure 3A**). However, the ratio of Foxp3^+^YFP^+^ versus Foxp3^+^YFP^-^ cells in *RhoA^Flox/Flox^Foxp3^YFP-Cre^*^/+^ female mice was reduced to ~ 0.40:1 (**Figure 3A**). The decrease in Foxp3^+^YFP^+^ cells in *RhoA^Flox/Flox^Foxp3^YFP-Cre^*^/+^ mice was associated with decreased proliferation and increased apoptosis (**Figures 3B, C**). The results suggest that RhoA-deficient Foxp3^+^YFP^+^ Treg cells are outcompeted by RhoA-proficient Foxp3^+^YFP^-^ Treg cells in *RhoA^Flox/Flox^Foxp3^YFP-Cre^*^/+^ mice. This may be explained by increased plasticity of RhoA-deficient Foxp3^+^YFP^+^ Treg cells that showed decreased expression of Foxp3 (**Figure 3D**) and increased expression of effector T cell cytokines IFN-γ and IL-4 (**Figure 3E**), compared with RhoA-proficient Foxp3^+^YFP^-^ Treg cells from the same mice. Plastic Treg cells may be impaired in their suppressive function (11). In support, plastic Foxp3^+^YFP^+^ Treg cells from *RhoA^Flox/Flox^Foxp3^YFP-Cre/+^* mice exhibited lower suppressive activity toward naïve T cell proliferation, compared to that from *RhoA^+/+^Foxp3^YFP-Cre^*^/+^ mice (**Figure 3F**). Thus, the plasticity of RhoA-deficient Treg cells in non-inflammatory *RhoA^Flox/Flox^Foxp3^YFP-Cre^*^/+^ female mice suggests that the plasticity of RhoA-deficient Treg cells in inflammatory *RhoA^Flox/Flox^Foxp3^YFP-Cre^* mice is not due to continuing inflammation but a cell-intrinsic effect.

**Figure 4:**
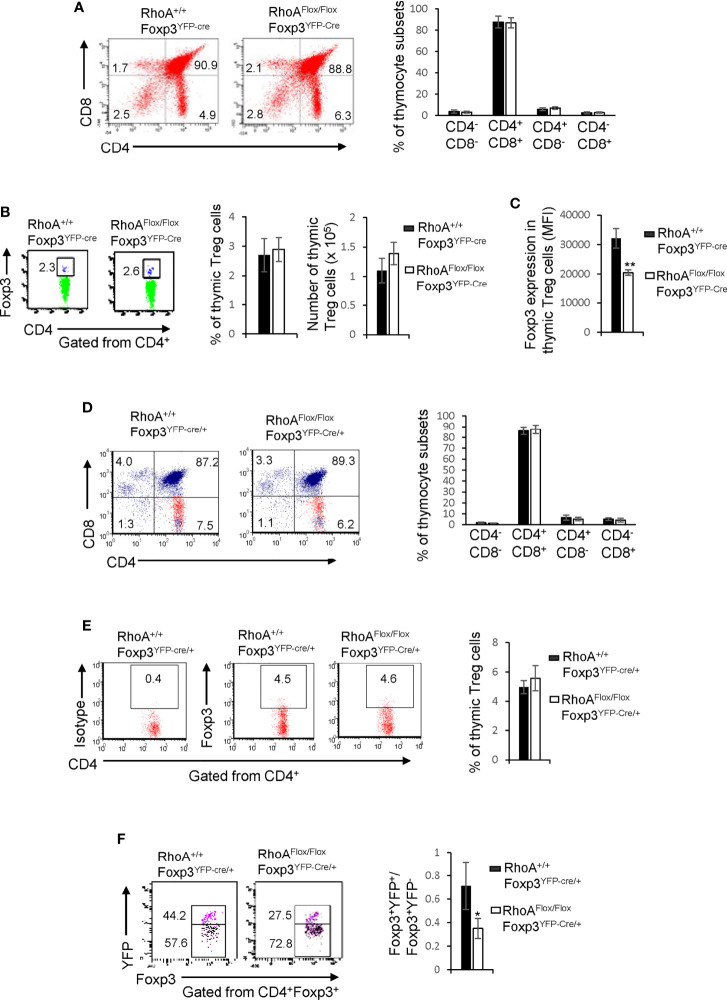
Homozygous *RhoA* deletion in Treg cells does not affect Treg cell development in the thymus but induces thymic Treg cell plasticity. **(A)** Left, representative flow cytogram of CD4 and CD8 staining in thymocytes from RhoA^+/+^Foxp3^YFP-Cre^ and RhoA^Flox/Flox^Foxp3^YFP-Cre^ mice. The numbers indicate percentages of CD4^-^CD8^-^, CD4^+^CD8^+^, CD4^+^ and CD8^+^ cells in corresponding quadrant. Right, average percentages of CD4^-^CD8^-^, CD4^+^CD8^+^, CD4^+^ and CD8^+^ cells. **(B)** Left, representative flow cytogram of Foxp3 staining in CD4^+^ thymocytes from RhoA^+/+^Foxp3^YFP-Cre^ and RhoA^Flox/Flox^Foxp3^YFP-Cre^ mice. The numbers indicate percentages of CD4^+^Foxp3^+^ thymic Treg cells. Middle, average percentages of CD4^+^Foxp3^+^ thymic Treg cells. Right, cell numbers of CD4^+^Foxp3^+^ thymic Treg cells. **(C)** The expression levels (MFI: mean fluorescence intensity) of Foxp3 in thymic Treg cells from RhoA^+/+^Foxp3^YFP-Cre^ and RhoA^Flox/Flox^Foxp3^YFP-Cre^ mice. **(D)** Left, representative flow cytogram of CD4 and CD8 staining in thymocytes from RhoA^+/+^Foxp3^YFP-Cre/+^ and RhoA^Flox/Flox^Foxp3^YFP-Cre/+^ female mice. The numbers indicate percentages of CD4^-^CD8^-^, CD4^+^CD8^+^, CD4^+^ and CD8^+^ cells in corresponding quadrant. Right, average percentages of CD4^-^CD8^-^, CD4^+^CD8^+^, CD4^+^ and CD8^+^ cells. **(E)** Left, representative flow cytogram of Foxp3 staining in CD4^+^ thymocytes from RhoA^+/+^Foxp3^YFP-Cre/+^ and RhoA^Flox/Flox^Foxp3^YFP-Cre/+^ female mice. The numbers indicate percentages of CD4^+^Foxp3^+^ thymic Treg cells. Right, average of percentages of CD4^+^Foxp3^+^ thymic Treg cells. **(F)** Left, representative flow cytogram of Foxp3^+^YFP^+^ and Foxp3^+^YFP^-^ cells in CD4^+^Foxp3^+^ thymic Treg cells from RhoA^+/+^Foxp3^YFP-Cre/+^ and RhoA^Flox/Flox^Foxp3^YFP-Cre/+^ female mice. The numbers indicate percentages of Foxp3^+^YFP^+^ and Foxp3^+^YFP^-^ cells. Right, the ratio of Foxp3^+^YFP^+^ versus Foxp3^+^YFP^-^ cells. n = 3 mice. Data are representative of two independent experiments. Error bars indicate SD. *p < 0.05, **p < 0.01.

The impaired homeostasis and the plasticity of RhoA-deficient Treg cells could be an outcome of impaired Treg cell development in the thymus. In this context, we analyzed thymocyte development in *RhoA^+/+^Foxp3^YFP-Cre^* and *RhoA^Flox/Flox^Foxp3^YFP-Cre^* mice. We found that *RhoA^Flox/Flox^Foxp3^YFP-Cre^* mice showed comparable frequencies of CD4^-^CD8^-^, CD4^+^CD8^+^, CD4^+^CD8^-^, and CD4^-^CD8^+^ thymocytes to *RhoA^+/+^Foxp3^YFP-Cre^* mice (**Figure 4A**). The thymic Treg cells also remained unaltered in *RhoA^Flox/Flox^Foxp3^YFP-Cre^* mice (**Figure 4B**). However, Foxp3 expression in *RhoA^Flox/Flox^Foxp3^YFP-Cre^* thymic Treg cells was downregulated (**Figure 4C**). Similar to *RhoA^Flox/Flox^Foxp3^YFP-Cre^* mice, *RhoA^Flox/Flox^Foxp3^YFP-Cre^*^/+^ female mice had intact CD4^-^CD8^-^, CD4^+^CD8^+^, CD4^+^CD8^-^, CD4^-^CD8^+^ thymocytes (**Figure 4D**) and thymic Treg cells (**Figure 4E**). Nonetheless, the ratio of Foxp3^+^YFP^+^ versus Foxp3^+^YFP^-^ cells in the thymus of *RhoA^Flox/Flox^Foxp3^YFP-Cre^*^/+^ female mice was significantly decreased (**Figure 4F**). Collectively, these data suggest that RhoA deficiency does not affect overt thymocyte/thymic Treg cell development but induces thymic Treg cell plasticity. Hence, it appears that the altered peripheral Treg cell homeostasis is not due to impaired Treg cell development in the thymus. In contrast, the plasticity of peripheral Treg cells seems to be inherited from thymic Treg cells.

### Heterozygous *RhoA* Deletion in Treg Cells Does Not Cause Systemic Autoimmunity and Impair Treg Cell Homeostasis but Induces Treg Cell Plasticity and Anti-Tumor T Cell Immunity

**Figure 5:**
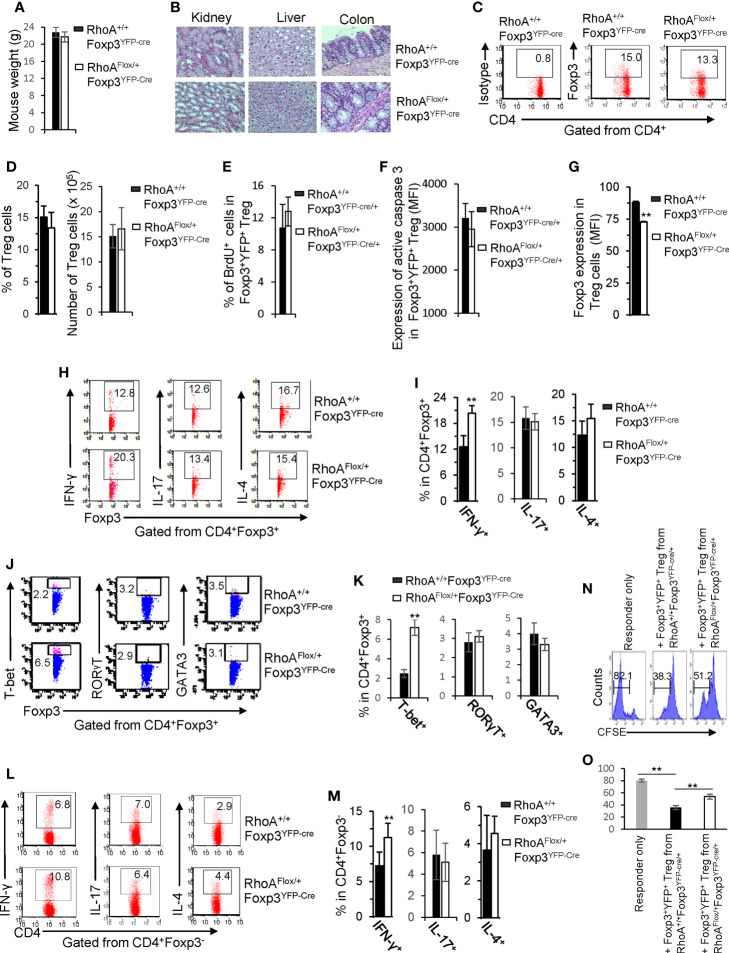
Heterozygous *RhoA* deletion in Treg cells induces Treg cell plasticity and increases CD4^+^ effector T cells but does not result in autoimmunity. **(A)** Body weight of RhoA^+/+^Foxp3^YFP-Cre^ and RhoA^Flox/+^Foxp3^YFP-Cre^ mice. **(B)** Images of H&E staining of the indicated organs. **(C)** Representative flow cytogram of Foxp3 staining in CD4^+^ cells from the spleen of RhoA^+/+^Foxp3^YFP-Cre^ and RhoA^Flox/+^Foxp3^YFP-Cre^ mice. The numbers indicate percentages of CD4^+^Foxp3^+^ Treg cells. **(D)** Left, average percentages of CD4^+^Foxp3^+^ Treg cells. Right, cell numbers of CD4^+^Foxp3^+^ Treg cells. **(E)** Proliferation of Foxp3^+^YFP^+^ Treg cells from RhoA^+/+^Foxp3^YFP-Cre/+^ and RhoA^Flox/+^Foxp3^YFP-Cre/+^ female mice. Percentages of Foxp3^+^YFP^+^ Treg cells incorporated with BrdU are shown. **(F)** Apoptosis of Foxp3^+^YFP^+^ Treg cells from RhoA^+/+^Foxp3^YFP-Cre/+^ and RhoA^Flox/+^Foxp3^YFP-Cre/+^ female mice. The expression levels (MFI: mean fluorescence intensity) of active caspase 3 in Foxp3^+^YFP^+^ Treg cells are shown. **(G)** The expression levels of Foxp3 in Treg cells from RhoA^+/+^Foxp3^YFP-Cre^ and RhoA^Flox/+^Foxp3^YFP-Cre^ mice. **(H)** Representative flow cytogram of IFN-γ, IL-17 and IL-4 staining in CD4^+^Foxp3^+^ Treg cells. The numbers indicate percentages of IFN-γ^+^, IL-17^+^ and IL-4^+^ Treg cells. **(I)** Average percentages of IFN-γ^+^, IL-17^+^ and IL-4^+^ Treg cells. **(J)** Representative flow cytogram of RORγT, T-bet and GATA3 staining in CD4^+^Foxp3^+^ Treg cells. The numbers indicate percentages of RORγT ^+^, T-bet^+^, and GATA3^+^ Treg cells. **(K)** Average percentages of RORγT ^+^, T-bet^+^, and GATA3^+^ Treg cells. **(L)** Representative flow cytogram of IFN-γ, IL-17 and IL-4 staining in CD4^+^Foxp3^-^ cells. The numbers indicate percentages of IFN-γ^+^CD4^+^, IL-17^+^CD4^+^ and IL-4^+^CD4^+^ effector T cells. **(M)** Average percentages of IFN-γ^+^CD4^+^, IL-17^+^CD4^+^ and IL-4^+^CD4^+^ effector T cells. **(N, O)** *In vitro* Treg suppressive activity. **(N)**, representative flow cytogram of CFSE dilution in T cells (responder). The numbers above the gating are percentages of cells low for CFSE, representing proliferating T cells. **(O)**, average percentages of cells low for CFSE. Foxp3^+^YFP^+^ Treg cells were obtained by flow cytometry sorting of CD4^+^YFP^+^ cells from the spleen of RhoA^+/+^Foxp3^YFP-Cre/+^ and RhoA^Flox/+^Foxp3^YFP-Cre/+^ female mice. **(A, C–O)** Error bars indicate SD of 5-6 mice **(A, C–M)** or triplicates **(N, O)**. Data are representative of two independent experiments. Error bars indicate SD. **p < 0.01. **(B)** Data are representative of 3 mice.

We next characterized *RhoA^Flox/+^Foxp3^YFP-Cre^* mice that bear heterozygous *RhoA* deletion in Treg cells. In contrast to homozygous *RhoA* deletion, heterozygous *RhoA* deletion did not lead to systemic autoimmunity, as evidenced by comparable mouse weight (**Figure 5A**) and tissue histopathology (**Figure 5B**) between *RhoA^Flox/+^Foxp3^YFP-Cre^* and *RhoA^+/+^Foxp3^YFP-Cre^* mice. Treg cell homeostasis was also unaffected by heterozygous *RhoA* deletion (**Figures 5C, D**), which is in line with intact proliferation and survival, as evidenced by comparable BrdU^+^ and active Caspase-3-expressing Foxp3^+^YFP^+^ Treg cells in *RhoA^Flox/+^Foxp3^YFP-Cre/+^* mice to that in *RhoA^+/+^Foxp3^YFP-Cre/+^* mice (**Figures 5E, F**). However, heterozygous *RhoA* deletion mimicked homozygous *RhoA* deletion in inducing Treg cell plasticity - it reduced the expression of Foxp3 (**Figure 5G**) and increased the expression of effector T cell cytokine IFN-γ and/or transcription factor T-bet in Treg (**Figures 5H–K**) and effector T cells (**Figures 5L, M**). Furthermore, in consistent with that plastic Treg cells may have dampened function (11), Foxp3^+^YFP^+^ Treg cells from *RhoA^Flox/+^Foxp3^YFP-Cre/+^* mice showed weakened suppressive activity toward naïve T cell proliferation, in comparison to that from *RhoA^+/+^Foxp3^YFP-Cre/+^* mice (**Figures 5N, O**).

**Figure 6:**
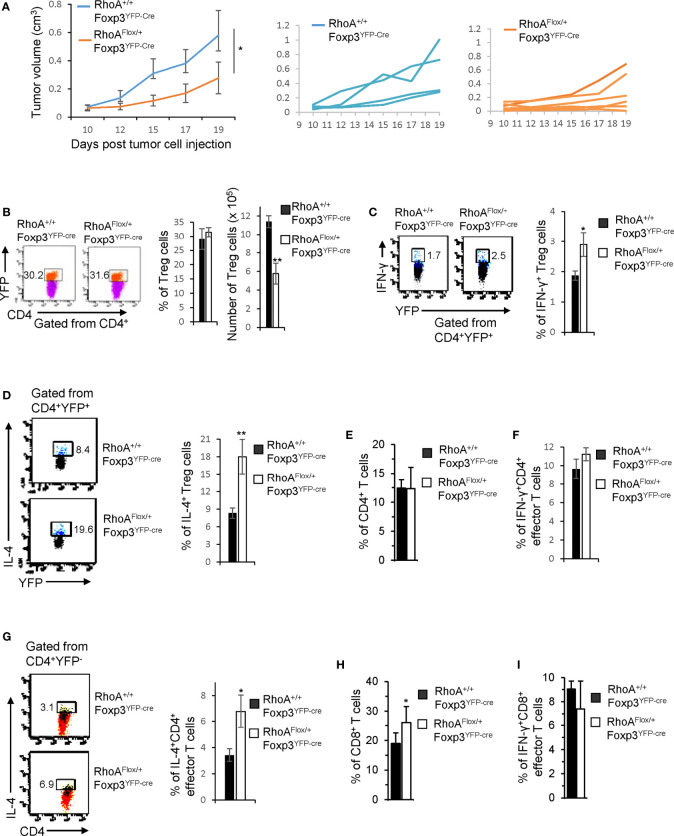
Heterozygous *RhoA* deletion in Treg cells inhibits tumor growth by promoting Treg cell plasticity. **(A)** Left, average tumor growth of MC38 mouse colon cancer cells in RhoA^+/+^Foxp3^YFP-Cre^ and RhoA^Flox/+^Foxp3^YFP-Cre^ mice. Middle, tumor growth in individual RhoA^+/+^Foxp3^YFP-Cre^ mice. Right, tumor growth in individual RhoA^Flox/+^Foxp3^YFP-Cre^ mice. **(B)** Left, representative flow cytogram of CD4^+^YFP^+^ cells from the tumors of RhoA^+/+^Foxp3^YFP-Cre^ and RhoA^Flox/+^Foxp3^YFP-Cre^ mice. The numbers indicate percentages of tumor-infiltrating CD4^+^YFP^+^ Treg cells. Middle, average percentages of tumor-infiltrating CD4^+^YFP^+^ Treg cells. Right, cell numbers of tumor-infiltrating CD4^+^YFP^+^ Treg cells. **(C)** Left, representative flow cytogram of IFN-γ staining in tumor-infiltrating CD4^+^YFP^+^ Treg cells. The numbers indicate percentages of tumor-infiltrating IFN-γ^+^ Treg cells. Right, average percentages of tumor-infiltrating IFN-γ^+^ Treg cells. **(D)** Left, representative flow cytogram of IL-4 staining in tumor-infiltrating CD4^+^YFP^+^ Treg cells. The numbers indicate percentages of tumor-infiltrating IL-4^+^ Treg cells. Right, average percentages of tumor-infiltrating IL-4^+^ Treg cells. **(E)** Percentages of tumor-infiltrating CD4^+^ effector T cells. **(F)** Percentages of tumor-infiltrating IFN-γ^+^CD4^+^ effector T cells. **(G)** Left, representative flow cytogram of IL-4 staining in tumor-infiltrating CD4^+^YFP^-^ effector T cells. The numbers indicate percentages of tumor-infiltrating IL-4^+^CD4^+^ effector T cells. Right, average percentages of tumor-infiltrating IL-4^+^CD4^+^ effector T cells. **(H)** Percentages of tumor-infiltrating CD8^+^ effector T cells. **(I)** Percentages of tumor-infiltrating IFN-γ^+^CD8^+^ effector T cells. n = 4-6 mice. Data are representative of two independent experiments. Error bars indicate SD. *p < 0.05, **p < 0.01.

While intact Treg cells promote tumor immune evasion (7), plastic Treg cells may invoke anti-tumor T cell immunity (32). To determine whether *RhoA^Flox/+^Foxp3^YFP-Cre^* Treg cells can be harnessed for tumor control, we inoculated *RhoA^Flox/+^Foxp3^YFP-Cre^* and *RhoA^+/+^Foxp3^YFP-Cre^* mice with MC38 mouse colon cancer cells. We found that tumor growth (measured by tumor volume) was markedly suppressed in *RhoA^Flox/+^Foxp3^YFP-Cre^* mice (**Figure 6A**). Although the frequencies of tumor-infiltrating Treg cells were not altered in *RhoA^Flox/+^Foxp3^YFP-Cre^* mice, their numbers were decreased (**Figure 6B**). There were more tumor-infiltrating IFN-γ^+^ and IL-4^+^ Treg cells in *RhoA^Flox/+^Foxp3^YFP-Cre^* mice than that in *RhoA^+/+^Foxp3^YFP-Cre^* mice (**Figures 6C, D**). Furthermore, IL-4-producing CD4^+^ effector T cells, but not total or IFN-γ-producing CD4^+^ effector T cells, were increased in the tumors in *RhoA^Flox/+^Foxp3^YFP-Cre^* mice (**Figures 6E–G**). Albeit IFN-γ-producing CD8^+^ effector T cells were unchanged, total CD8^+^ effector T cells were elevated in the tumors in *RhoA^Flox/+^Foxp3^YFP-Cre^* mice (**Figures 6H, I**). Taken together, these results suggest that *RhoA* heterozygosity inhibits Treg cell infiltration into tumors, induces tumor-infiltrating Treg cell plasticity and triggers T cell immunity against tumor growth.

## Discussion

In this study, we show that homozygous *RhoA* deletion in Treg cells causes early, fatal inflammatory disorders, suggesting that RhoA is a bona fide gatekeeper of immune tolerance. The autoimmune responses in *RhoA^Flox/Flox^Foxp3^YFP-Cre^* mice are associated with increased effector T cells that presumably result from decreased Treg cells and/or increased Treg cell plasticity. The decrease in Treg cells is likely due to impaired proliferation and survival but not development in the thymus. The plasticity of *RhoA^Flox/Flox^Foxp3^YFP-Cre^* Treg cells appears to be a cell-autonomous phenomenon but could be an inherited phenotype from thymic Treg cells. *RhoA^Flox/Flox^Foxp3^YFP-Cre^* Treg cell plasticity is manifested by Foxp3 downregulation and effector T cell reprogramming. As reduced Foxp3 expression is known to convert Treg cells to effector T cells (30, 33), at least a proportion of the increased CD4^+^ effector T cells in *RhoA^Flox/Flox^Foxp3^YFP-Cre^* mice could have been derived from *RhoA^Flox/Flox^Foxp3^YFP-Cre^* Treg cells. Given that Treg cells expressing effector T cell markers are implicated in autoimmune diseases such as inflammatory bowel diseases (34), the effector T cell reprogramming in *RhoA^Flox/Flox^Foxp3^YFP-Cre^* Treg cells might have contributed to effector T cell functions and thus the inflammatory diseases in *RhoA^Flox/Flox^Foxp3^YFP-Cre^* mice.

In contrast to homozygous deletion of *RhoA*, heterozygous deletion of *RhoA* does not appear to cause autoimmunity and perturb Treg cell homeostasis. However, similar to *RhoA* homozygosity, *RhoA* heterozygosity induces Treg cell plasticity. We note that while all of effector T cell cytokines IFN-γ, IL-17 and IL-4 are upregulated in Treg cells and CD4^+^ effector T cells in *RhoA^Flox/Flox^Foxp3^YFP-Cre^* mice, only IFN-γ is upregulated in Treg and CD4^+^ effector T cells in *RhoA^Flox/+^Foxp3^YFP-Cre^* mice, suggesting that RhoA dosages fine-tune Treg cell plasticity. Importantly, *RhoA^Flox/+^Foxp3^YFP-Cre^* Treg cells are capable of invoking anti-tumor T cell immunity. Nonetheless, unlike steady-state *RhoA^Flox/+^Foxp3^YFP-Cre^* mice, tumor-bearing *RhoA^Flox/+^Foxp3^YFP-Cre^* mice show increased expression of IL-4, in addition to IFN-γ, in tumor-infiltrating Treg cells. Furthermore, IL-4, but not IFN-γ, was elevated in intratumoral CD4^+^ effector T cells. Thus, it seems that the manifestations of Treg cell plasticity are context-dependent. As IL-4 has been reported to be able to suppress cancer progression (35), we postulate that IL-4 produced by tumor-infiltrating Treg cells and CD4^+^ effector T cells, IFN-γ produced by tumor-infiltrating Treg cells, and non-IFN-γ effector molecules (e.g. TNF-α, granzyme B and/or perforin) produced by tumor-infiltrating CD8^+^ T cells collectively inhibit tumor growth in *RhoA^Flox/+^Foxp3^YFP-Cre^* mice.

Treg cell plasticity may dampen their suppressive function (11). In support, plastic Treg cells bearing homozygous or heterozygous *RhoA* deletion are impaired in their suppression of naïve T cell proliferation. As these Treg cells are derived from *RhoA^Flox/Flox^Foxp3^YFP-Cre/+^* or *RhoA^Flox/+^Foxp3^YFP-Cre/+^* mice that contain RhoA-proficient Treg cells and show no overt inflammation, our findings suggest that RhoA promotes Treg suppressive function in a cell-intrinsic manner. Of note, *RhoA^Flox/Flox^Foxp3^YFP-Cre^* Treg cells upregulate their functional markers CTLA-4, GITR and PD-1. We envision that these Treg cells undergo functional exhaustion, reminiscent of LKB1-deficient Treg cells (36).

In summary, our findings suggest that graded RhoA expression distinguishes tumor immunity from autoimmunity. We demonstrate a proof-of-concept that pharmacological titration of RhoA to induce Treg cell plasticity without perturbation of their homeostasis may elicit anti-tumor T cell immunity without causing systemic autoimmune diseases.

## Data Availability Statement

The raw data supporting the conclusions of this article will be made available by the authors, without undue reservation.

## Ethics Statement

The animal study was reviewed and approved by Animal Care and Use Committee, Cincinnati Children’s Hospital Medical Center.

## Author Contributions

KK designed and performed the research and analyzed the data. J-QY and VM performed the research and analyzed the data. PN and YL performed the research. YZ designed the research, analyzed the data, and contributed vital new reagents or analytical tools. FG designed the research, analyzed the data, contributed vital new reagents or analytical tools, and wrote the paper. All authors contributed to the article and approved the submitted version.

## Funding

This work was supported in part by grants from the National Institutes of Health (R01GM108661 to FG, R56 HL141499 to FG, and R01CA234038 to FG and YZ).

## Conflict of Interest

The authors declare that the research was conducted in the absence of any commercial or financial relationships that could be construed as a potential conflict of interest.

## Publisher’s Note

All claims expressed in this article are solely those of the authors and do not necessarily represent those of their affiliated organizations, or those of the publisher, the editors and the reviewers. Any product that may be evaluated in this article, or claim that may be made by its manufacturer, is not guaranteed or endorsed by the publisher.

## References

[1] Annunziato F Romagnani C Romagnani S. The 3 Major Types of Innate and Adaptive Cell-Mediated Effector Immunity. J Allergy Clin Immunol (2015) 135:626–35. doi: 10.1016/j.jaci.2014.11.001 25528359

[2] Finn OJ. A Believer's Overview of Cancer Immunosurveillance and Immunotherapy. J Immunol (2018) 200:385–91. doi: 10.4049/jimmunol.1701302 PMC5763509 29311379

[3] Uqba Khan U Ghazanfar H. T Lymphocytes and Autoimmunity. Int Rev Cell Mol Biol (2018) 341:125–68. doi: 10.1016/bs.ircmb.2018.05.008 30262031

[4] Ohkura N Kitagawa Y Sakaguchi S. Development and Maintenance of Regulatory T Cells. Immunity (2013) 38:414–23. doi: 10.1016/j.immuni.2013.03.002 23521883

[5] Dang EV Barbi J Yang HY Jinasena D Yu H Zheng Y. Control of T(H)17/T(reg) Balance by Hypoxia-Inducible Factor 1. Cell (2011) 146:772–84. doi: 10.1016/j.cell.2011.07.033 PMC3387678 21871655

[6] Gomez-Rodriguez J Wohlfert EA Handon R Meylan F Wu JZ Anderson SM. Itk- Mediated Integration of T Cell Receptor and Cytokine Signaling Regulates the Balance Between Th17 and Regulatory T Cells. J Exp Med (2014) 211:529–43. doi: 10.1084/jem.20131459 PMC3949578 24534190

[7] Byrne WL Mills KH Lederer JA O'Sullivan GC. Targeting Regulatory T Cells in Cancer. Cancer Res (2011) 71:6915–20. doi: 10.1158/0008-5472.CAN-11-1156 PMC4287207 22068034

[8] Shitara K Nhshikawa H. Regulatory T Cells: A Potential Target in Cancer Immunotherapy. Ann NY Acad Sci (2018) 1417:104–15. doi: 10.1111/nyas.13625 29566262

[9] Colamatteo A Carbone F Bruzzaniti S Galgani M Fusco C Maniscalco GT. Molecular Mechanisms Controlling Foxp3 Expression in Health and Autoimmunity: From Epigenetic to Post-Translational Regulation. Front Immunol (2020) 10:3136. doi: 10.3389/fimmu.2019.03136 32117202 PMC7008726

[10] Cortez JT Montauti E Shifrut E Gatchalian J Zhang Y Shaked O. CRISPR Screen in Regulatory T Cells Reveals Modulators of Foxp3. Nature (2020) 582:416–20. doi: 10.1038/s41586-020-2246-4 PMC7305989 32499641

[11] Takahashi R Nishimoto S Muto G Sekiya T Tamiya T Kimura A. SOCS1 Is Essential for Regulatory T Cell Functions by Preventing Loss of Foxp3 Expression as Well as IFN-{Gamma} and IL-17A Production. J Exp Med (2011) 208:2055–67. doi: 10.1084/jem.20110428 PMC3182063 21893603

[12] Tnimov Z Guo Z Gambin Y Nguyen UTT Wu YW Abankwa D. Quantitative Analysis of Prenylated RhoA Interaction With Its Chaperone, RhoGDI. J Biol Chem (2012) 287:26549–62. doi: 10.1074/jbc.M112.371294 PMC3410996 22628549

[13] Nobes CD Hall A. Rho, Rac, and Cdc42 GTPases Regulate the Assembly of Multimolecular Focal Complexes Associated With Actin Stress Fibers, Lamellipodia, and Filopodia. Cell (1995) 81:53–62. doi: 10.1016/0092-8674(95)90370-4 7536630

[14] Lin R Cerione RA Manor D. Specific Contributions of the Small GTPases Rho,Rac, and Cdc42 to Dbl Transformation. J Biol Chem (1999) 274:23633–41. doi: 10.1074/jbc.274.33.23633 10438546

[15] Guo F Zheng Y. Involvement of Rho Family GTPases in p19Arf- and P53-Mediated Proliferation of Primary Mouse Embryonic Fibroblasts. Mol Cell Biol (2004) 24:1426–38. doi: 10.1128/mcb.24.3.1426-1438.2004 PMC321455 14729984

[16] Zohn IM Campbell SL Khosravi-Far R Rossman KL Der CJ. Rho Family Proteins and Ras Transformation: The RHOad Less Traveled Gets Congested. Oncogene (1998) 17:1415–38. doi: 10.1038/sj.onc.1202181 9779988

[17] Olson MF Ashworth A Hall A. An Essential Role for Rho, Rac, and Cdc42 GTPases in Cell Progression Through G1. Science (1995) 269:1270–2. doi: 10.1126/science.7652575 7652575

[18] Rougerie P Largeteau Q Megrelis L Carrette F Lejeune T Toffali L. Fam65b Is a New Transcriptional Target of FOXO1 That Regulates RhoA Signaling for T Lymphocyte Migration. J Immunol (2013) 190:748–55. doi: 10.4049/jimmunol.1201174 23241886

[19] del Pozo MA Vicente-Manzanares M Tejedor R Serrador JM Sanchez-Madrid F. Rho GTPases Control Migration and Polarization of Adhesion Molecules and Cytoskeletal ERM Components in T Lymphocytes. Eur J Immunol (1999) 29:3609–20. doi: 10.1002/(SICI)1521-4141(199911)29:11<3609::AID-IMMU3609>3.0.CO;2-S 10556816

[20] Heasman SJ Carlin LM Cox S Ng T Ridley AJ. Coordinated RhoA Signaling at the Leading Edge and Uropod Is Required for T Cell Transendothelial Migration. J Cell Biol (2010) 190:553–63. doi: 10.1083/jcb.201002067 PMC2928012 20733052

[21] Vielkind S Gallagher-Gambarelli M Gomez M Hinton HJ Cantrell DA. Integrin Regulation by RhoA in Thymocytes. J Immunol (2005) 175:350–7. doi: 10.4049/jimmunol.175.1.350 15972668

[22] Mou F Praskova M Xia F Van Buren D Hock H Avruch J. The Mst1 and Mst2 Kinases Control Activation of Rho Family GTPases and Thymic Egress of Mature Thymocytes. J Exp Med (2012) 209:741–59. doi: 10.1084/jem.20111692 PMC3328371 22412158

[23] Corre I Gomez M Vielkind S Cantrell DA. Analysis of Thymocyte Development Reveals Thath Te GTPase RhoA Is a Positive Regulator of T Cell Receptor Responses *In Vivo*. J Exp Med (2001) 194:903–14. doi: 10.1084/jem.194.7.903 PMC2193481 11581313

[24] Henning SW Galandrini R Hall A Cantrell DA. The GTPase Rho Has a Critical Regulatory Role in Thymus Development. EMBO J (1997) 16:2397–407. doi: 10.1093/emboj/16.9.2397 PMC1169840 9171353

[25] Galandrini R Henning SW Cantrell DA. Different Functions of the GTPase Rho in Prothymocytes and Late Pre-T Cells. Immunity (1997) 7:163–74. doi: 10.1016/s1074-7613(00)80519-1 9252129

[26] López-Posadas R Fastancz P Carmen Martínez-Sánchez LD Julia Panteleev-Ivlev J Thonn V Kisseleva T. Inhibiting PGGT1B Disrupts Function of RHOA, Resulting in T-Cell Expression of Integrin α4β7 and Development of Colitis in Mice. Gastroenterology (2019) 157:1293–309. doi: 10.1053/j.gastro.2019.07.007 31302143

[27] Yang JQ Kalim KW Li Y Zhang S Hinge A Filippi MD. RhoA Orchestrates Glycolysis for Th2 Cell Differentiation and Allergic Airway Inflammation. J Allergy Clin Immunol (2016) 137:231–45. doi: 10.1016/j.jaci.2015.05.004 PMC4684821 26100081

[28] Yang JQ Kalim KW Li Y Zheng Y Guo F. Ablation of RhoA Impairs Th17 Cell Differentiation and Alleviates House Dust Mite-Triggered Allergic Airway Inflammation. J Leukoc Biol (2019) 106:1139–51. doi: 10.1002/JLB.3A0119-025RRR PMC7747217 31260596

[29] Melendez J Stengel K Zhou X Chauhan BK Debidda M Andreassen P. RhoA GTPase Is Dispensable for Actomyosin Regulation But Is Essential for Mitosis in Primary Mouse Embryonic Fibroblasts. J Biol Chem (2011) 286:15132–7. doi: 10.1074/jbc.C111.229336 PMC3083211 21454503

[30] Kalim KW Yang JQ Li Y Meng Y Zheng Y Guo F. Reciprocal Regulation of Glycolysis- Driven Th17 Pathogenicity and Treg Stability by Cdc42. J Immunol (2018) 200:2313–26. doi: 10.4049/jimmunol.1601765 PMC5860966 29440353

[31] Komatsu N Okamoto K Sawa S Nakashima T Oh-hora M Kodama T. Pathogenic Conversion of Foxp3+ T Cells Into TH17 Cells in Autoimmune Arthritis. Nat Med (2014) 20:62–8. doi: 10.1038/nm.3432 24362934

[32] Wei J Long L Yang K Guy C Shrestha S Chen Z. Autophagy Enforces Functional Integrity of Regulatory T Cells by Coupling Environmental Cues and Metabolic Homeostasis. Nat Immunol (2016) 17(3):277–85. doi: 10.1038/ni.3365 PMC4755832 26808230

[33] Wan YY Flavell RA. Regulatory T-Cell Functions Are Subverted and Converted Owing to Attenuated Foxp3 Expression. Nature (2007) 445:766–70. doi: 10.1038/nature05479 17220876

[34] Ueno A Ghosh A Hung D Li J Jijon H. Th17 Plasticity and Its Changes Associated With Inflammatory Bowel Disease. World J Gastroenterol (2015) 21:12283–95. doi: 10.3748/wjg.v21.i43.12283 PMC4649113 26604637

[35] Li S Liu M Do MH Chou C Stamatiades EG Nixon BG. Cancer Immunotherapy *via* Targeted TGF-β Signalling Blockade in T_H_ Cells. Nature (2020) 587:121–5. doi: 10.1038/s41586-020-2850-3 PMC8353603 33087933

[36] Yang K Blanco DB Neale G Vogel P Avila J Clish CB. Homeostatic Control of Metabolic and Functional Fitness of Treg Cells by LKB1 Signalling. Nature (2017) 548(7669):602–6. doi: 10.1038/nature23665 PMC5804356 28847007

